# Supplementary material for: Conformational distortion in a fibril-forming oligomer arrests alpha-Synuclein fibrillation and minimizes its toxic effects
Source: Commun Biol. 2021 May 3;4:518. doi: 10.1038/s42003-021-02026-z (PMC8093279; doi:10.1038/s42003-021-02026-z)
Supplement: Supplementary file 1 — Supplementary Information [file 42003_2021_2026_MOESM1_ESM.pdf]

Supplementary Information

Conformational distortion in a fibril-forming oligomer arrests alpha-Synuclein fibrillation and minimizes its toxic effects

Ritobrita Chakraborty,[a] Sandip Dey,[a] Pallabi Sil,[b] Simanta Sarani Paul,[c] Dipita Bhattacharyya,[d] Anirban Bhunia,[d] Jayati Sengupta,[*, a] Krishnananda Chattopadhyay[*, a]

[a] Structural Biology and Bioinformatics Division, CSIR-Indian Institute of Chemical Biology, 4, Raja SC Mullick Road, Kolkata 700032, India

[b] Present address: Department of Physics, University of Alberta, Edmonton, Canada

[c]Department of Medicine, Centre for Prion and Protein folding disease, University of Alberta, Edmonton, Canada

[d] Department of Biophysics, Bose Institute- Centenary Campus, P-1/12 C.I.T. Scheme VII-M, Kolkata - 700054, India.

[*] Corresponding author: krish@iicb.res.in, jayati@iicb.res.in

Running title: Structural understanding of a non-toxic oligomer of alpha-Synuclein

Keywords: Amyloid fibrils, Cryo-electron microscopy, Toxic oligomer, Greek key motif, Heme-induced inhibition, Molecular mechanism

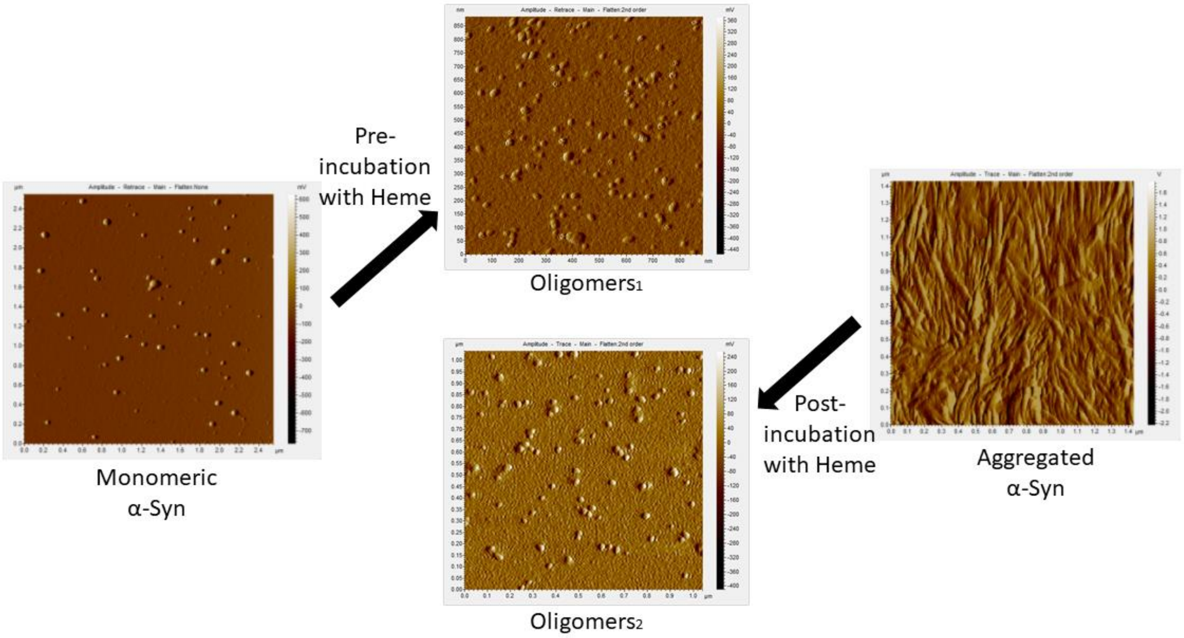

Supplementary Figure 1: **Schematic representation explaining the formation of oligomers$_1$ and oligomers$_2$ upon heme addition.** AFM images explain the nomenclature of oligomers$_1$ and oligomers$_2$ based upon the stage of heme addition.

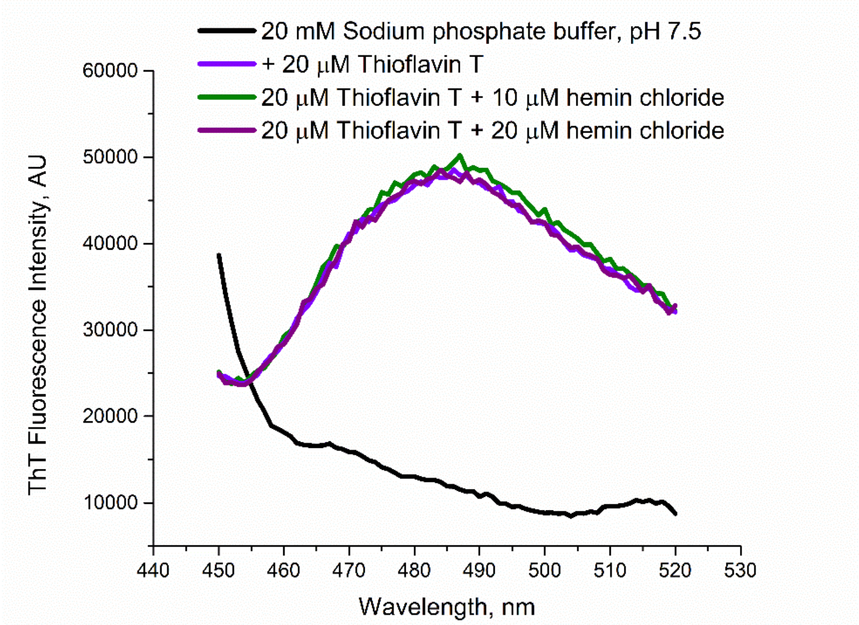

Supplementary Figure 2: **Effect of heme on Thioflavin T fluorescence.** The concentration of heme used in our studies (which is 8 μM) does not have a quenching effect on the fluorescence of 20 μM Thioflavin T. Both heme and ThT were dissolved in sodium phosphate buffer, pH 7.5.

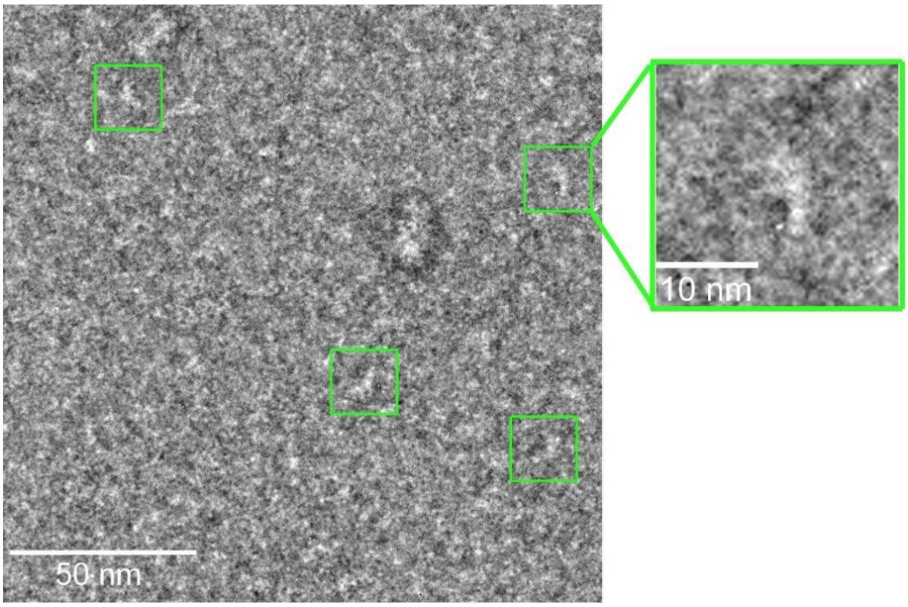

Supplementary Figure 3: **TEM image of oligomer$_2$.** Magnified view of heme-treated oligomers$_2$ observed using negative-stain TEM. The oligomers$_2$ are depicted within the green boxes. The inset shows a single elongated ‘comma’-shaped particle. This is in good agreement with the cryo-EM density map, shown in Figure 4, main text.

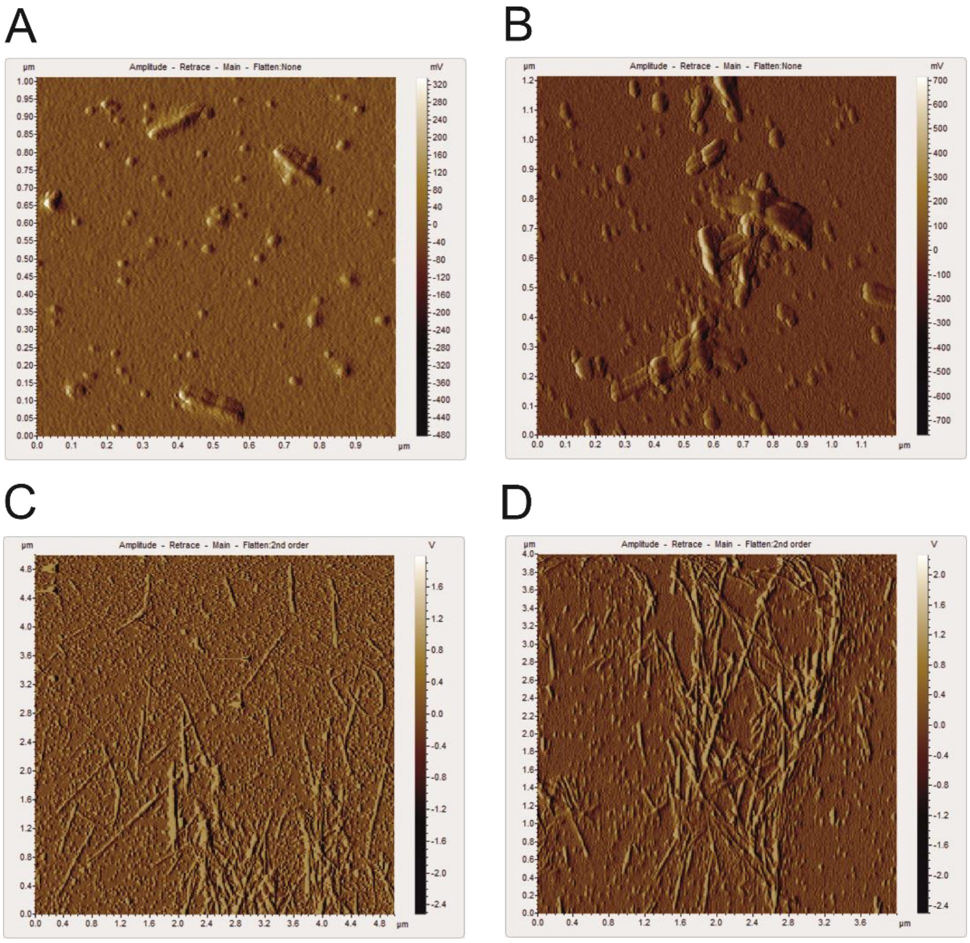

Supplementary Figure 4: **Fibrillation of α-Syn.** AFM micrographs depicting the structurally heterogeneous aggregates. The aggregates formed after (A) 24 h, (B) 36 h, (C) 48 h, and (D) 60 h of aggregation under constant agitation at 37ºC in the absence of heme correspond to the toxicity profile depicted in Figure 2D, main manuscript.

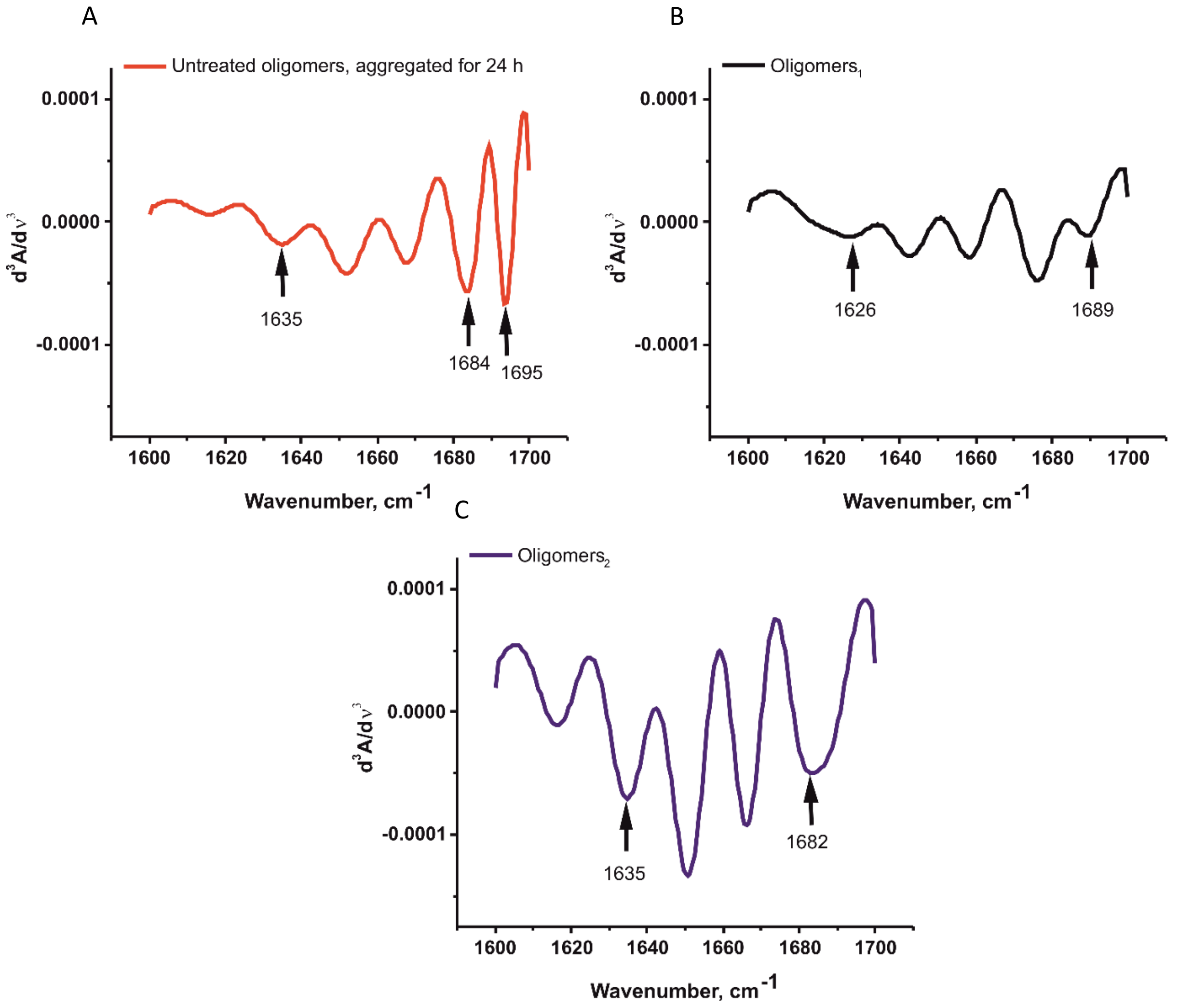

Supplementary Figure 5: **FTIR second derivative spectra of the α-Syn oligomers**. FTIR second derivative spectra of the α-Syn oligomers show the positions of overlapping peaks. In case of the untreated oligomers (A), the presence of a marked band at 1695 cm^{-1} denotes the presence of antiparallel β sheet structure. This 1695 cm^{-1} band is noticeably absent in the spectra for the heme-treated oligomers$_1$ (B) and oligomers$_2$ (C). Additionally, the band at ~1685 cm^{-1} (also denotes antiparallel β sheet) is also reduced in case of the heme-treated oligomers. This is quantified by deconvoluting the IR spectra (refer Figure 3D, main text) where we find that the heme-treated oligomers show an obvious reduction in antiparallel β sheet content from 12% (untreated oligomers) to 2% (oligomers$_1$) and 5% (oligomers$_2$).

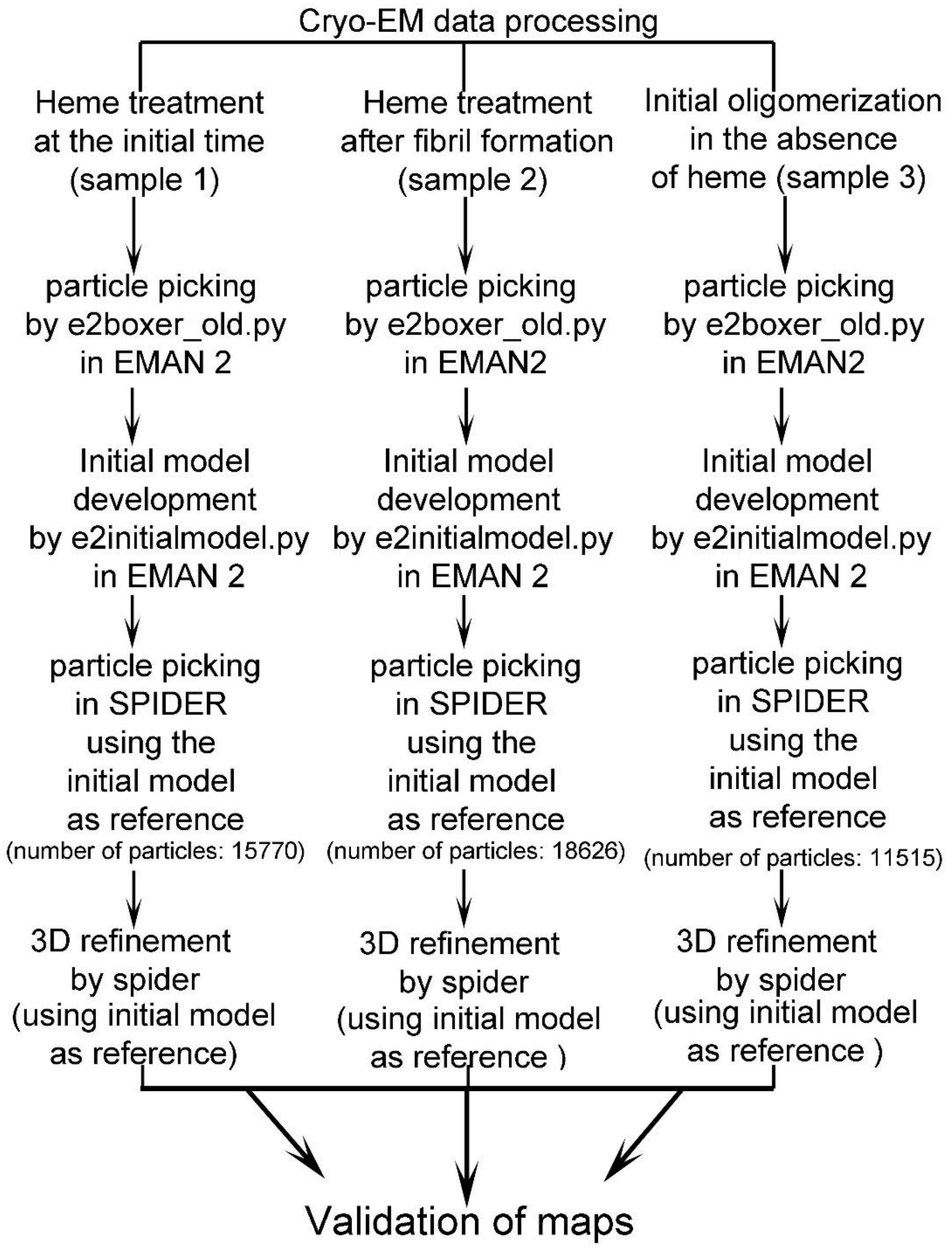

Supplementary Figure 6: **Cryo-EM image processing.** Flowchart describing the sequence used for image processing so as to generate the three cryo-EM maps.

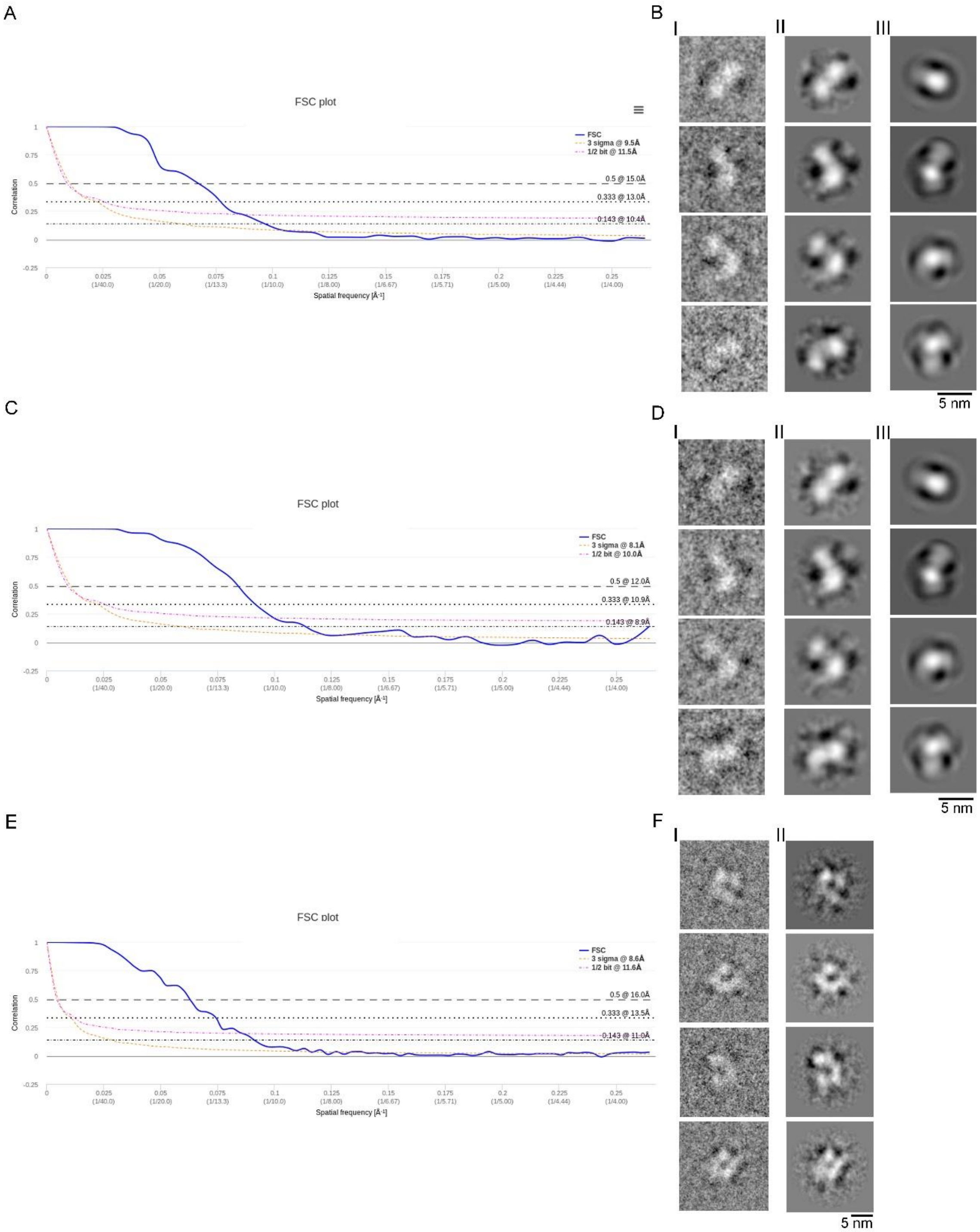

Supplementary Figure 7: **Analysis of cryo-EM image processing of the three datasets.** Analysis of the cryo-EM image processing of three datasets.FSC plots (A, C and E) show resolutions 15 Å, 12Å and 16Å (0.5 FSC cutoff) of (A) sample 1: oligomers$_1$ formed when α-Syn is pre-treated with heme; (C) sample 2: oligomers$_2$formed upon post-treatment of α-Syn with heme; and (E) sample 3: semi-annular oligomer formed in the absence of heme, respectively. (B, D, and F) show comparison of 2D averages for samples 1, 2, and 3, respectively. Different views (I) of 2D class averages of particles and projection of final maps (II) are compared. 2D projections of density map created from the structure **PDB 2N0A** (depicted as tetrameric) are shown in III (B, D).

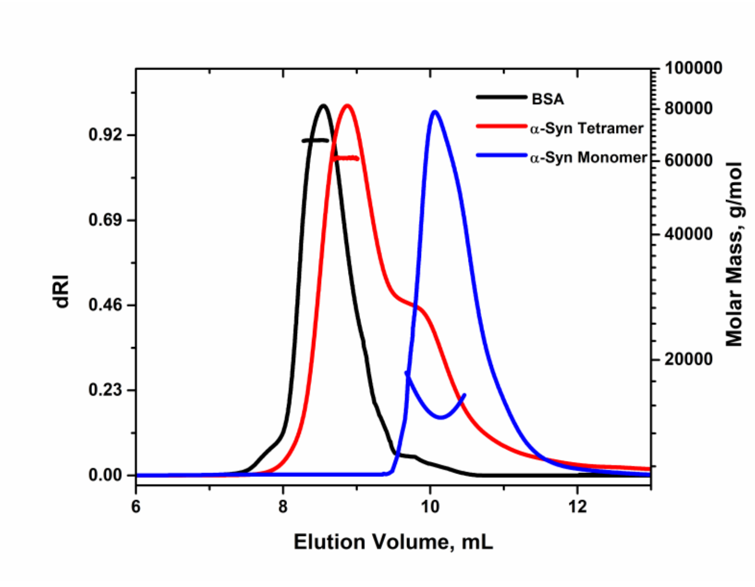

Supplementary Figure 8: **SEC-MALS analyses of α-Syn.** SEC-MALS analyses of Bovine Serum Albumin (BSA, 66.4 g/mol, in black), monomeric α-Synuclein (14.46 g/mol, in blue) and heme-treated oligomers$_1$ (60.2 g/mol, in red). The continuous lines correspond to the differential refractive indices (660 nm, left ordinate axis), and horizontal line segments correspond to the calculated molar masses for the corresponding peaks (right ordinate axis).

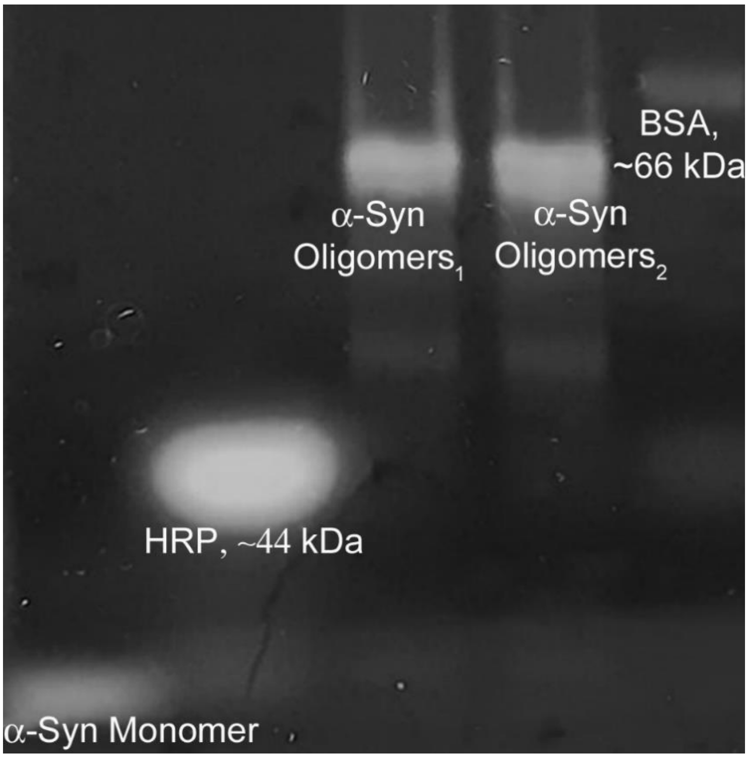

Supplementary Figure 9: **Native PAGE analyses of α-Syn.** A 10 % Native PAGE of AlexaFluor488-tagged α-Syn monomer (MW ~15 kDa, first lane from left), 5-TAMRA, SE-tagged horseradish peroxidase (HRP, MW ~44 kDa, lane 2), AlexaFluor488-tagged α-Syn oligomer$_1$ (lane 3), AlexaFluor488-tagged α-Syn oligomer$_2$ (lane 4), 5-TAMRA, SE-tagged

Bovine Serum Albumin (BSA, 66.4 kDa, lane 5). The monomer migrated at ~15 kDa as has been reported before[1-3] while the oligomers$_1$ and oligomers$_2$ migrated at ~60 kDa. These fluorescently labeled proteins were also subjected to FCS to correlate the FCS r_H values with their observed migration pattern.

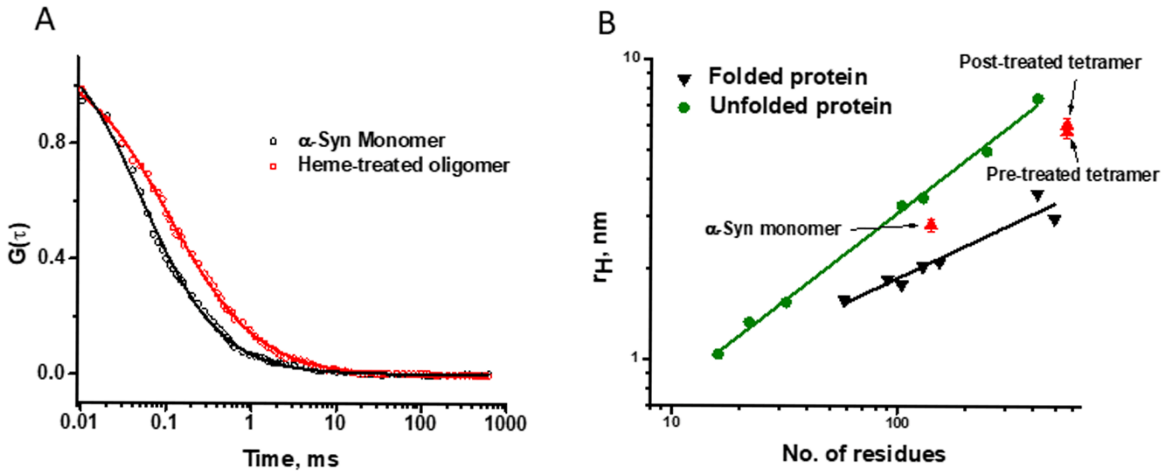

Supplementary Figure 10: **FCS analyses of α-Syn oligomerization.** (A) FCS correlation curves of α-Syn monomer (open red circles) plotted with heme-treated oligomers (black circles) indicate an increase in the diffusion time (and hence the $r_{H\ Exp}$) of the heme-treated oligomers. The data were fit to a single-component diffusion model (solid lines). (B) Plot of the $\log_e$ of the hydrodynamic radius (r_H) versus the $\log_e$ of the number of residues in the polypeptide chain. The line fitted to these data for the native folded proteins (black) has a slope of 0.29±0.02 and a y-axis intercept of 1.56±0.1, while that fitted to the chemically denatured protein (green) data has a slope of 0.57±0.02 and a y-axis intercept of 0.79±0.07. Literature data have been used for the folded and chemically denatured proteins[4] while we employed FCS to calculate the r_H of the pre-treated oligomers (oligomers$_1$), post-treated oligomers (oligomers$_2$). Both oligomers have a $r_{H,\ FCS}$ that lies between that of natively-folded and unfolded proteins.

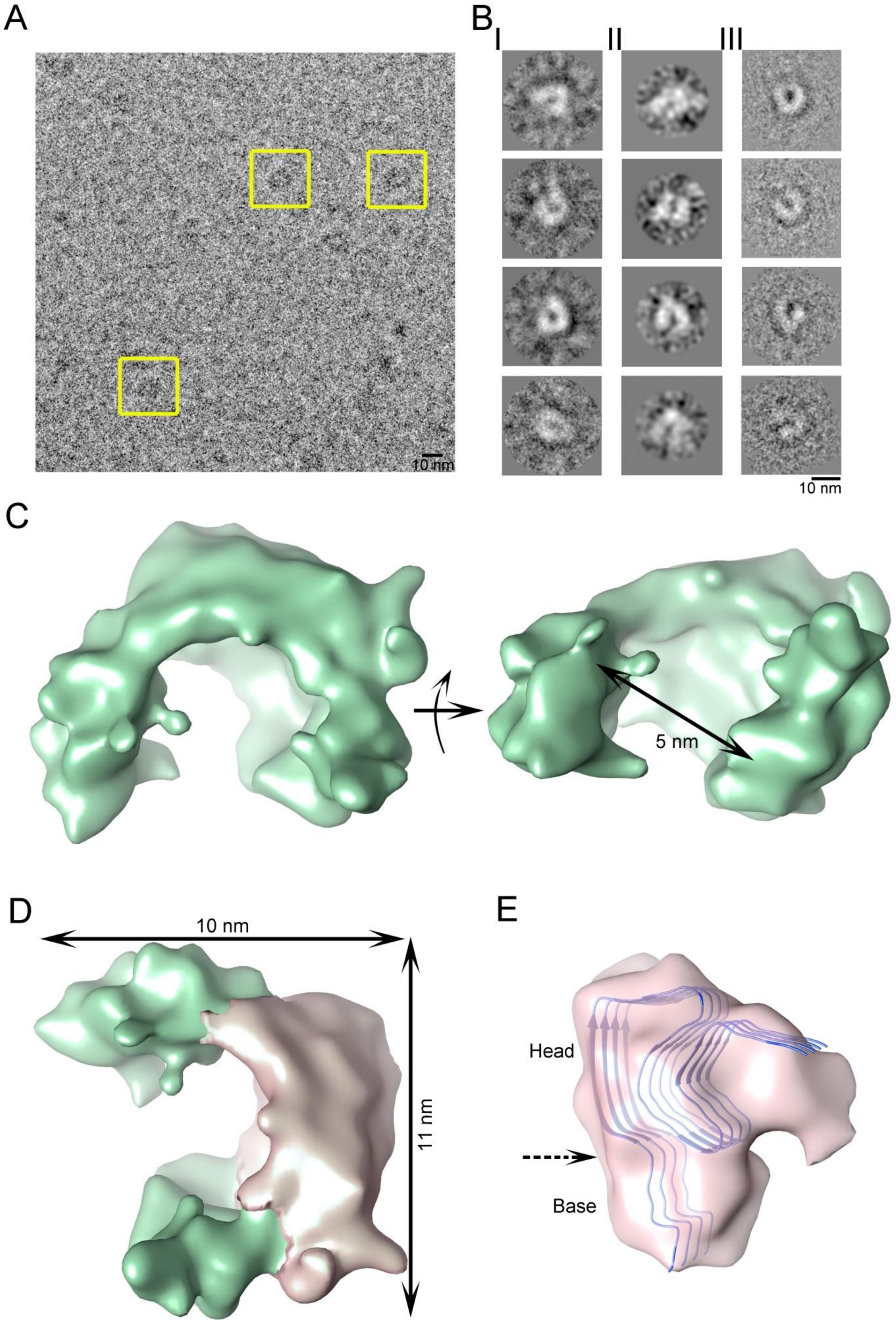

1

Supplementary Figure 11: **Early α-Syn oligomer formation without heme treatment.** Cryo-EM study of (A-C) the oligomersformed ~24 h of aggregation in the absence of heme. (A) Micrograph depicting distribution of a prominant oligomeric species (yellow boxes) within a heterogeneous population. (B I-III) Reference-free 2D class averages generated in Xmipp, RELION and EMAN2, respectively, showing the semi-annular shape of the oligomer. (C) Two views of the 3D cryo-EM density map of the semi-annular oligomer of α-Syn formed in the absence of heme.Apparently, this form might be a precursor of previously reported annular oligomeric forms. (D) One of the segment of the density map is overlaid (pink, segmentation performed using Chimera). (E) Docking of α-Syn Greek key motif (tetrameric unit, blue cartoon) into the isolated density segment (semitransparent pink) shows accommodation of the coordinates without any requirement of distortion at the junction of ‘head’ and ‘base’ (marked by dotted arrow) indicating that the tetrameric Greek key motif is most likely the fundamental unit for α-Syn oligomerization.

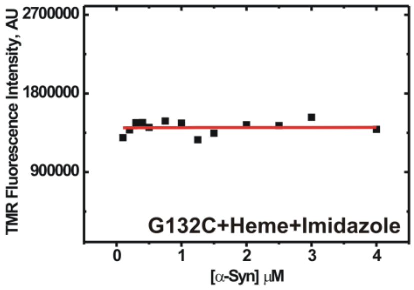

Supplementary Figure 12: **Involvment of histidine residue in heme-binding.** Heme shows no binding to TMR-5-maleimide-tagged α-Syn G132C in presence of excess (10 mM) imidazole, suggesting the probable role of histidine residues in the binding interactions.

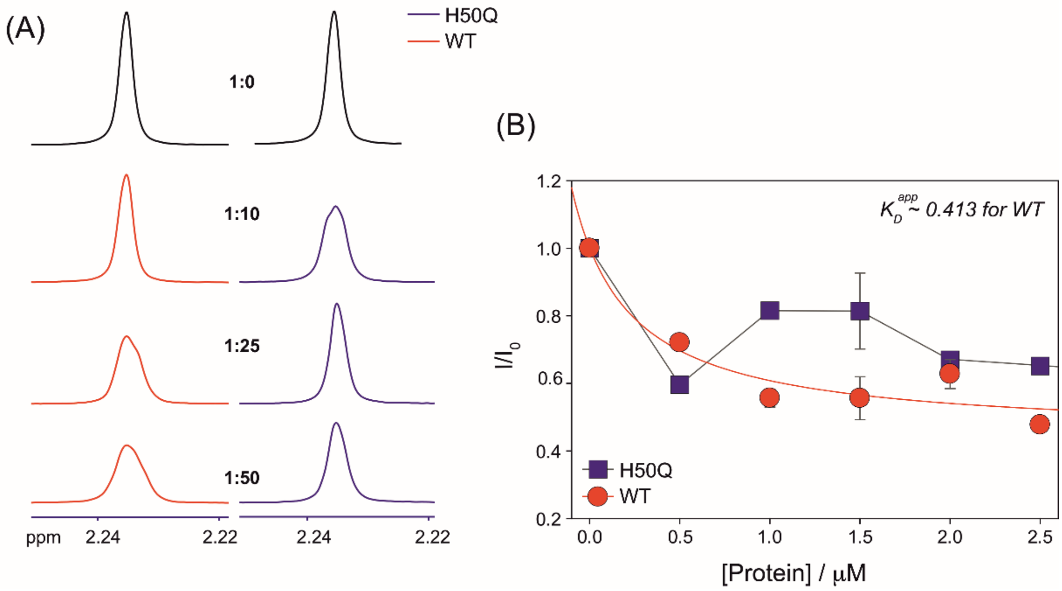

Supplementary Figure 13: **Interaction of heme with Histidine 50 of α-Syn.** (A) 1H NMR peak intensity decay of 0.05 μM heme for the sharp singlet at around 2.235ppm upon increasing concentrations of the WT (red) and the H50Q variant (blue). (B) The ratios of the peak intensities with respect to the free ligand (I/I_0) show a gradual decrease upon WT interaction that fits a binding equation resulting in an apparent $K_D$app value of ~0.413 μM. H50Q protein shows a non-specific interaction with heme.

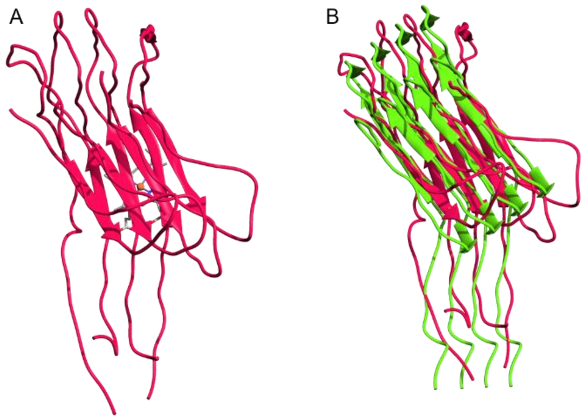

Supplementary Figure 14: **Dynamics of heme-arrested α-Syn oligomer.** (A) A well-equilibrated and stable end-simulation structure shows the presence of the distortion upon heme-binding that (B) matches with the cryo-EM fitted model of 2N0A (refer Figure 7, main text).

**Condition**	**Band position (cm^{-1})**	**% Area**	**Assignment**
α-Syn oligomer, aggregated for 24 h hours	1693.65	1.31	β -Sheet (High component), antiparallel
	1683.56	10.48	β -Sheet (High component), antiparallel
	1667.82	24.90	β -turn
	1651.97	22.43	Loops/Disorder
	1634.72	36.63	β -Sheet
	1615.72	4.24	Extended/Side chains

α-Syn oligomer$_1$, pre-treatment with heme for 24 h	1689.77	2.21	β -Sheet (High component), antiparallel
	1676.12	26.97	β -turn
	1658.37	25.88	Loops
	1642.76	21.16	Disorder/Extended
	1626.82	23.72	β -Sheet
α-Syn oligomer$_2$, post-treatment with heme, after 48 h of aggregation	1683.28	5.14	β -Sheet (High component), antiparallel
	1666.19	26.43	β -turn
	1650.88	33.35	Disorder/Extended
	1635.03	28.07	β -Sheet
	1616.33	7.00	Extended/Side chains

Supplementary Table 1: FTIR-based evaluation of secondary structure content during the fibrillation process for α-Syn oligomers with heme introduced at selected time-points.

Data collection and processing	Heme arrested off-pathway oligomer of alpha-synuclein<br>(EMDB ID: EMD-31004)
Magnification	×79365
Voltage (kV)	300
Electron exposure (e–/Å^2)	20
Defocus range (μm)	1-5

Pixel size (Å)	1.89
Symmetry imposed	C1
Initial particle images (no.)	15770 (Manually Picked)
Final particle images (no.)	15770
Map resolution (Å)	10.4
FSC threshold	0.143
Map resolution range (Å)	8-13
Initial model used (PDB code)	2N0A (selected tetrameric form to fit into the cryo-EM map using two segments (head and body) as rigid bodies). *

Supplementary Table 2: Cryo-EM data collection and map details.

Supplementary References:

1. Hoffmann, A.-C. *et al.* Extracellular aggregated alpha synuclein primarily triggers lysosomal dysfunction in neural cells prevented by trehalose. *Sci Rep* **9**, 544-544, doi:10.1038/s41598-018-35811-8 (2019).
2. Chen, S. W. *et al.* Structural characterization of toxic oligomers that are kinetically trapped during α-synuclein fibril formation. *Proc. Natl. Acad. Sci. U. S. A.* **112**, E1994 (2015).
3. Bopardikar, M. *et al.* Triphala inhibits alpha-synuclein fibrillization and their interaction study by NMR provides insights into the self-association of the protein. *RSC Advances* **9**, 28470-28477, doi:10.1039/C9RA05551G (2019).
4. Wilkins, D. K. *et al.* Hydrodynamic radii of native and denatured proteins measured by pulse field gradient NMR techniques. *Biochemistry* **38**, 16424-16431, doi:10.1021/bi991765q (1999).
