# Supplementary material for: Conformational distortion in a fibril-forming oligomer arrests alpha-Synuclein fibrillation and minimizes its toxic effects
Source: Commun Biol. 2021 May 3;4:518. doi: 10.1038/s42003-021-02026-z (PMC8093279; doi:10.1038/s42003-021-02026-z)
Supplement: Supplementary file 6 — Description of Additional Supplementary Files [file 42003_2021_2026_MOESM6_ESM.pdf]

Description of Additional Supplementary Files

File name: Supplementary Movie 1

Description: Distortion observed at the junction of the head and the base of the heme-stabilized mace oligomer (in orange) overlaid on a tetrameric unit of the solid-state NMR-derived Greek key model (PDB 2N0A, in blue).

File name: Supplementary Data 1

Description: Source data for all graphs.
